# Progression of subclinical and clinical cardiovascular disease in a UK SLE cohort: the role of classic and SLE-related factors

**DOI:** 10.1136/lupus-2018-000267

**Published:** 2018-11-17

**Authors:** Sahena Haque, Sarah Skeoch, Chadi Rakieh, Helena Edlin, Yasmeen Ahmad, Pauline Ho, Rachel Gorodkin, M Yvonne Alexander, Ian N Bruce

**Affiliations:** 1 Arthritis Research UK Centre for Epidemiology, Centre for Musculoskeletal Research, Faculty of Biology Medicine and Health, The University of Manchester, Manchester Academic Health Science Centre, Manchester, UK; 2 Department of Rheumatology, Wythenshawe Hospital, Manchester University NHS Foundation Trust, Manchester, UK; 3 The Kellgren Centre for Rheumatology, NIHR Manchester Biomedical Research Centre, Manchester University Hospitals NHS Foundation Trust, Manchester Academic Health Science Centre, Manchester, UK; 4 Department of Vascular Surgery, Manchester University NHS Foundation Trust, Manchester, UK; 5 Peter Maddison Rheumatology Centre, Llandudno Hospital, Manchester, UK; 6 Centre for Bioscience, School of Healthcare Science, Manchester Metropolitan University, Manchester, UK

**Keywords:** atherosclerosis, Cardiovascular Disease, ultrasonography

## Abstract

**Objectives:**

We aimed to describe the rate and determinants of carotid plaque progression and the onset of clinical cardiovascular disease (CVD) in a UK SLE cohort.

**Methods:**

Female patients with SLE of white British ancestry were recruited from clinics in the North-West of England and had a baseline clinical and CVD risk assessment including measurement of carotid intima–media thickness (CIMT) and plaque using B-mode Doppler ultrasound. Patients were followed up (>3.5 years after baseline visit) and had a repeat carotid Doppler to assess progression of plaque and CIMT. Clinical CVD events between visits were also noted.

**Results:**

Of 200 patients with a baseline scan, 124 (62%) patients had a second assessment at a median (IQR) of 5.8 (5.2–6.3) years follow-up. New plaque developed in 32 (26%) (4.5% per annum) patients and plaque progression was observed in 52 (41%) patients. Factors associated with plaque progression were older age (OR 1.13; 95%  CI 1.06 to 1.20), anticardiolipin (OR 3.36; 1.27 to 10.40) and anti-Ro (OR 0.31; 0.11 to 0.86) antibodies. CVD events occurred in 7.2% over 5.8 years compared with 1.0% predicted using the Framingham risk score (p<0.001). Higher triglycerides (OR 3.6; 1.23 to 10.56), cyclophosphamide exposure ‘ever’ (OR 16.7; 1.46 to 63.5) and baseline Systemic Lupus International Collaborating Clinics damage index score (OR 9.62; 1.46 to 123) independently predicted future CVD events.

**Conclusion:**

Accelerated atherosclerosis remains a major challenge in SLE disease management. A more comprehensive approach to CVD risk management taking into account disease factors such as severity and anticardiolipin antibody status may be necessary to improve CVD outcomes in this high-risk population.

## Introduction

The impact of accelerated atherosclerosis on morbidity and mortality in SLE has been well documented over the past four decades.^1 2^ Many questions remain unanswered, particularly relating to risk stratification and factors influencing the initiation and progression of atherosclerosis. While it is clear that traditional cardiovascular disease (CVD) risk factors play an important role,^3^ several studies have demonstrated that disease-specific factors are also important and as a result, traditional risk prediction models perform poorly in SLE.^4^ The exact contribution of inflammatory burden, renal disease and a procoagulant state in the context of long-term corticosteroid and immunosuppressant therapy can be difficult to evaluate. Studying clinical complications alone requires large long-term studies and therefore only a few studies have attempted to study predictors of actual atherosclerotic events in SLE.^3 5–7^ Several modalities have been used to measure subclinical atherosclerosis in SLE and approximately 30%–40% of patients have evidence of subclinical lesions by a range of methods.^8–10^ High-resolution B-mode Doppler ultrasound has been widely used to measure both carotid intima–media thickness (CIMT) and carotid plaque. Large general population studies have used CIMT and found an association with classic cardiovascular risk factors such as hypertension, hyperlipidaemia and family history of coronary heart disease (CHD)^11–13^ and also demonstrated CIMT to be an independent predictor of CHD.^14 15^ Within SLE, we and others have found carotid plaque in 29%–37% of patients compared with 15%–22% of controls.^16^ The excess of carotid plaque in SLE is particularly striking in those under 55 years old.^16 17^ There are only a few follow-up studies in patients with SLE, but these have shown progression of carotid plaque, which in turn predicted future CVD events.^18 19^ Factors that have been associated with plaque progression in SLE included homocysteine, higher C3 complement and immunosuppressive use.^18 19^ The aim of this study was to describe the rate and determinants of carotid atherosclerosis progression and the development of clinical CVD events in a UK SLE cohort.

## Methods

Our baseline cohort was assembled between 2002 and 2005 as previously described.^16^ This cohort was re-contacted and invited back for a further assessment at a minimum of 3.5 years after their baseline visit. We made several attempts to contact patients no longer under follow-up in their original clinic and/or who have moved address during the interim period. For those who died, we noted the cause of death from their clinic records and/or after discussion with their primary treating physician. The cohort consisted of female patients of white British ancestry^16^ who were at least 18 years old and fulfilled four or more 1997 American College of Rheumatology (ACR) criteria for SLE.^20^ Patients who fulfilled three criteria for SLE in the absence of any alternative diagnosis were also included, as previously described.^16^ All patients gave written informed consent and the study was approved by the Central Manchester Local Research Ethics Committee.

At baseline and at follow-up, patients had a clinical interview and examination to collect demographic information, family history, lifestyle factors, SLE disease status as well as information on traditional cardiovascular risk factors and CVD events (personal history from patient), that is, myocardial infarction, angina, cerebrovascular event, transient ischaemic attack (TIA), coronary intervention and peripheral vascular disease. Cardiovascular risk factors were defined as previously described (see also table 1) In addition, we defined metabolic syndrome according to the 2009 definition described in the Joint Interim Statement from the International Diabetes Federation Task Force on Epidemiology and Prevention and interested partners.^21^

**Table 1:** Description of 200 patients with SLE at the baseline study

Baseline factor	
Age at diagnosis: mean (SD) years	36.4 (11.8)
Disease duration: mean (SD) years	11.7 (9.4)
Previous cerebrovascular event: n (%)	16 (8)
Previous coronary event: n (%)	7 (3.5)
Peripheral vascular disease: n (%)	1 (0.5)
Ever ANA positive: n (%)	188 (94)
Ever dsDNA positive: n (%)	115 (57.5)
Ever aCL or LAC positive: n (%)	73 (37)
Antiphospholipid syndrome: n (%)	21 (10.5)
Current steroid therapy: n (%)	106 (53)
SLEDAI-2K: median (IQR)	1 (0–4)
SLICC damage index: median (IQR)	0 (0–4)
Current antimalarial therapy: n (%)	105 (52.5)
Current immunosuppressive therapy: n (%)	74 (37)
Postmenopausal: n (%)	94 (47)
Current smoker: n (%)	40 (20)
Ex-smoker: n (%)	57 (28.5)
Family history of premature CHD: n (%)	53 (26.5)
Total cholesterol: median (IQR) mmol/L	5.1 (4.3–6.0)
Fasting plasma glucose: median (IQR) mmol/L	4.6 (4.3–4.9)
Systolic blood pressure: median (IQR) mm Hg	126 (116–140)
Previous or current hypertension: n (%)	83 (41.5)
Diabetes (%)	6 (3.0)
BMI: median (IQR) kg/m^2^	26.0 (23.2–30.1)

Hypertension: blood pressure of >140/90 or current treatment with antihypertensive drug. Hypercholesterolemia: total cholesterol >5.2 mmol/L or LDL cholesterol >3.2 mmol/L or on therapy. Family history of premature CVD: MI, angina or sudden death in a first-degree relative male <55 years or female <65 years. Diabetes mellitus: fasting plasma glucose >7.0 mmol/L or current diabetic therapy.

aCL, anticardiolipin antibody; BMI, Body Mass Index; CHD, coronary heart disease; CVD, cardiovascular disease; LAC, lupus anticoagulant; MI, myocardial infarction; SLEDAI, Systemic Lupus Erythematosus Disease Activity Index; SLICC, Systemic Lupus International Collaborating Clinics.

This requires three or more of the following five criteria to be present: (1) elevated waist circumference (>80 cm for Europid women), (2) elevated triglycerides (≥1.7 mmol/L), (3) reduced HDL cholesterol (<1.3 mmol/L in women), (4) elevated blood pressure (≥130/85 mm Hg) or drug therapy for hypertension, and (5) elevated fasting glucose (≥5.6 mmol/L) or drug therapy for hyperglycaemia.^21^

We also collected information on SLE features and disease activity (Systemic Lupus Erythematosus Disease Activity Index - SLEDAI 2000 edition—SLEDAI-2K)^22^ and damage (ACR/Systemic Lupus International Collaborating Clinics damage index—SDI^23^). We defined ‘renal disease’ as having any one or more of persistent proteinuria (>500 mg/day), otherwise unexplained microscopic haematuria, chronic renal insufficiency, nephrotic syndrome or any class of lupus nephritis diagnosed on biopsy. Details of current and previous SLE therapy as well as other current medications were recorded. Steroid exposure was documented as any previous exposure, average daily dose (mg/day over the past 6 months), and duration of current and previous courses. In participants with no prior cardiovascular disease, we estimated the 5-year percentage risk of cardiovascular events using the Framingham risk equation.^24^

Carotid examinations were undertaken by one of two vascular technicians on the day of the clinical assessments, one of whom had performed the original scans but both were blinded to the baseline carotid examination results at time of the follow-up scan. CIMT and plaque were quantified as previously described.^16^ Briefly, the right and left common carotid artery (CCA), carotid bulb and the first 1.5 cm of the internal and external carotid arteries were examined in longitudinal and cross-sectional planes using the Philips HDI 5000. Intima–media thickness (IMT) was measured as previously described and validated.^25^ Measurements were made at the time of scanning, in a longitudinal plane at a point of maximum thickness on the far wall of the CCA along a 1 cm section of the artery proximal to the carotid bulb. Measurements were repeated three times on each side, unfreezing the image on each occasion and relocating the maximal IMT, and the average of six measurements were then used to calculate the mean IMT. Carotid plaque was defined if two of the following three conditions were met: (1) a distinct area of protrusion >50% compared with the surrounding area into the vessel lumen, (2) increased echogenicity than the adjacent boundaries and (3) IMT >0.15 cm 26. Plaque burden was estimated using the plaque index, a summary score of number and size of plaque as previously described.^25^ A good level of agreement was observed between technicians (mean difference −0.001, 95% limits of agreement −0.0098 to 0.0078).

All data were analysed using STATA V.13.1 statistical software. Comparisons were made by means of a two-sample t-test for normally distributed continuous variables and by χ^2^ analysis for categorical variables. For non-normally distributed variables, non-parametric tests were determined using Kruskal-Wallis rank test for categorical variables and Spearman’s correlation coefficients for continuous variables. Two-sided p values of less than 0.05 were considered to be significant. Age-adjusted linear and logistic regression analyses were used to determine the association between baseline risk factors and progression in CIMT, carotid plaque and incident clinical cardiovascular events. Standardised coefficients were used to assess the strength of associations between variables. A backward stepwise multivariable model which included baseline age, follow-up period and variables that had a significant relationship in the univariate analysis or could logically be a confounder was performed with a threshold for significance at p value <0.2 to assess the best models to predict plaque progression (defined as increase in plaque index including those with no plaque at baseline who developed plaque), CIMT progression and clinical CVD events.

## Results

Two hundred patients were assessed at baseline and 124 (62%) were contactable and agreed to return for assessment at a second timepoint. The median (IQR) time between visits for these 124 patients was 5.8 (5.2–6.3) years. The median (IQR) baseline age and disease duration was 49 (44–56) and 11 (4–18) years, respectively. Baseline clinical and serological features of those followed or lost to follow-up are summarised in table 2.^16^ Seventy-six (38%) of the baseline cohort did not participate in the follow-up study. Forty-five (23%) patients had a change of address or could not be contacted; 21 (10.5%) declined further participation and of these, one had developed throat cancer, one had bladder cancer and another had a stroke; the others declined for social reasons (caring for relatives, child care and employment). Ten patients (5%) died during the follow-up period; causes of death included malignancy (four in total; cervical, intracerebral, lung and liver), ruptured aortic aneurysm (n=1), cerebral haemorrhage (n=1) and gastrointestinal haemorrhage (n=1). The cause of death was unknown in three patients. Apart from higher triglycerides and lower systolic blood pressure at baseline, those who did not return did not differ significantly with respect to SLE features, therapy exposures or classic CVD risk factors to those who had a return visit (table 1).

**Table 2:** Baseline features of patients with SLE followed up compared with patients with SLE lost to follow-up (p>0.05 deemed non-significant (NS)) and characteristics at follow-up

Cohort characteristics	At baseline			Patients with SLE followed up N=124
	Patients with SLE lost to follow-up N=76	Patients with SLE followed up N=124	P values	
Age: median (IQR) years	45 (37–54)	49 (44–57)	NS	55 (50–62)
Disease duration: median (IQR) years	7 (4–15)	11 (4–18)	NS	17 (10–25)
Total cholesterol: mean (SD) mmol/L	5.3 (1.4)	5.1 (1.1)	NS	4.49 (1.96)
HDL cholesterol: mean (SD) mmol/L	1.68 (0.54)	1.65 (0.47)	NS	1.58 (1.43)
LDL cholesterol: mean (SD) mmol/L	2.70 (1.21)	2.76 (0.87)	NS	3.12 (1.13)
Triglyceride levels: mean (SD) mmol/L	1.37 (0.09)	1.16 (0.57)	0.01	1.18 (0.60)
Systolic blood pressure: mean (SD) mm Hg	125 (19)	132 (19)	0.03	131 (20.1)
Hypertension (%)	29	32	NS	41
Metabolic syndrome (%)	30.1	30.6	NS	–
Fasting glucose: mean (SD) mmol/L	4.7 (0.87)	4.6 (0.77)	NS	4.6 (0.85)
Smoking ever (%)	54	45	NS	45
Cardiovascular disease (%)	14	9.7	NS	16
Family history of CHD (%)	28	26	NS	26
Body Mass Index (kg/m^2^): mean (SD)	27 (6)	27 (6)	NS	28 (5.8)
Renal disease (%)	21.1	13	NS	18
SDI: median (IQR)	0 (0–2)	0 (0–1)	NS	1 (0–2)
SLEDAI: median (IQR)	0 (0–3)	1 (0–4)	NS	1 (0–4)
Anticardiolipin antibody positive (%)	28	32	NS	
Ro antibody positive (%)	34.2	37.1	NS	
La antibody positive (%)	10.9	17.7	NS	
DsDNA antibody positive (%)	59.	56	NS	
Antimalarial therapy (%)	77	86.	NS	
Cyclophosphamide therapy (%)	18	10	NS	

CHD, coronary heart disease; SDI, Systemic Lupus International Collaborating Clinics damage index; SLEDAI.

### Progression of carotid atherosclerosis

**Figure 1:**
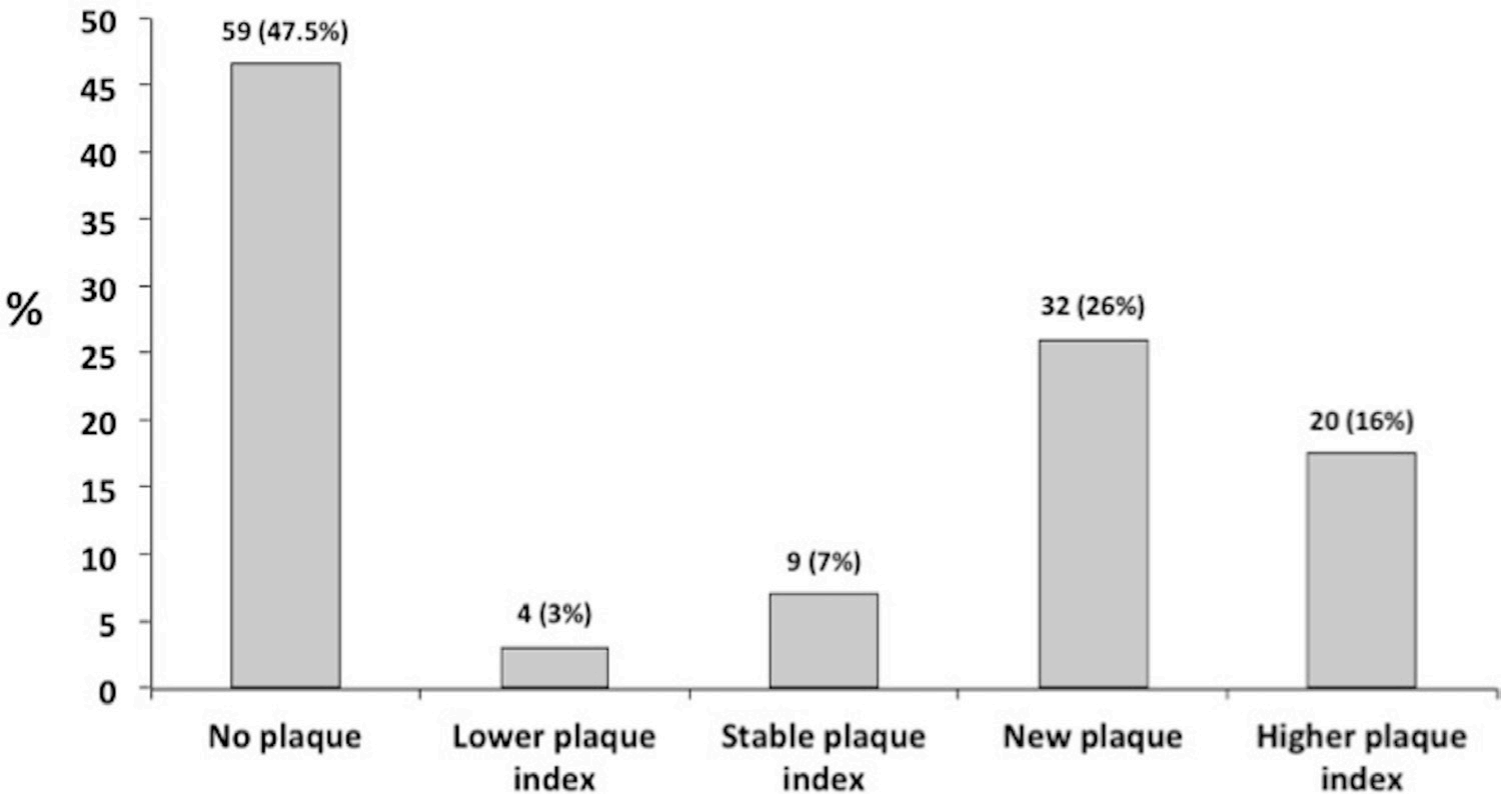
Summary of plaque at follow-up.

Table 3 summarises changes in carotid plaque and CIMT over time. At baseline, 34/124 (27%) patients had at least one carotid plaque and by follow-up, 63/124 (50%) patients had at least one plaque. The change in carotid plaque status is summarised in figure 1. Almost half of patients (59/124; 47.6%) had no plaque at either time point. Thirty-two patients (26%) free of plaque at baseline developed a new plaque at the second assessment. Of those with plaque at baseline, 20 (17.5%) had an increased plaque index, 9 (7%) patients had a stable plaque index over time and 4 (3.2%) had a lower plaque index at the follow-up assessment. Fifty-two (41%) patients had evidence of plaque progression, defined as either new plaque or increase in plaque index.

**Table 3:** Rate of carotid intima–media thickness (CIMT) and plaque progression in SLE

Follow-up time: mean (SD) years	5.72 (0.89)
Baseline plaque prevalence	345/124 (28%)
Follow-up plaque prevalence	63/124 (50%)
New plaque onset	32/124 (26%)
Plaque change/year	4.5%
Baseline CIMT: median (IQR) cm	0.05 (0.04–0.06)
Follow-up CIMT: median (IQR) cm	0.06 (0.05, 0.07)
CIMT change/year: mean (SD) cm/year	0.002 (0.001)

CIMT overall showed progression between visits (table 3); however, we noted that eight (7%) patients had regression of CIMT at follow-up.

In univariable analysis, baseline age was associated with both plaque and CIMT progression and therefore all analyses were adjusted for age and disease duration. Factors associated with plaque progression are summarised in table 4. The percentage of 5-year cardiovascular risk at baseline was significantly associated with plaque progression even after adjustment for disease duration (OR 1.51; 95% CI 1.11 to 2.05). Of note, positive Ro and La antibodies were both negatively associated with plaque progression (OR 0.27; 0.10 to 0.75 and OR 0.31; 0.11 to 0.86, respectively). In contrast, anticardiolipin (aCL) antibody status was associated with an increased risk of plaque progression (OR 3.64; 1.27 to 10.40). In a multivariable analysis, a positive aCL remained independently associated with plaque progression (OR 3.14; 1.10 to 9.01), and this was robust when we retained classic risk factors in the model (OR 3.57; 1.23 to 10.56).

**Table 4*:** Univariable analysis of baseline factors associated with progression of carotid atherosclerosis and clinical cardiovascular events (CVEs)

Baseline factors	Plaque progression		CIMT progression		CVEs	
	Age-adjusted OR	95% CI	Age-adjusted β (SE)	P values	Age-adjusted OR	95% CI
Prior CVEs	1.63	0.145 to 18.27	0.0059 (0.0035)	0.69	**4.89**	**1.15 to 20.65**
Disease duration (years)*	1.00	0.95 to 1.05	−0.08090 (0.00009)	0.39	1.04	0.97 to 1.09
Total cholesterol (mmol/L)*	0.90	0.617 to 1.31	−**0.19442** (**0.0008**)	**0.03**	0.93	0.51 to 1.6
Hypercholesterolemia	4.31	0.86 to 21.59	0.07482 (0.00283)	0.46	1.48	0.22 to 5.86
LDL cholesterol (mmol/L)*	0.93	0.58 to 1.60	−0.07829 (2.9×10^−4^)	0.41	0.61	0.28 to 1.34
Triglycerides (mmol/L)*	1.25	0.61 to 2.55	−0.06203 (2.0×10^−4^)	0.18	**2.92**	**1.3 to 6.46**
Systolic blood pressure (mm Hg)*	1.01	0.99 to 1.05	−0.17274 (0.00005)	0.06	1.038	0.96 to 1.12
Hypertension	1.12	0.43 to 3.23	−0.04238 (0.00203)	0.53	**6.19**	**1.5 to 25.47**
Fasting glucose (mmol/L)*	0.72	0.30 to 1.70	−0.10982 (0.00125)	0.19	1.00	
Type 2 diabetes	0.63	0.03 to 13.77	−0.01446 (0.00754)	0.10	No events in diabetic group	NA
Smoking ever	1.67	0.0.68 to 3.89	0.05302 (0.00191)	0.55	0.64	0.18 to 2.24
Family history of CHD	0.45	0.17 to 1.18	−0.07237 (0.00215)	0.39	1.27	0.34 to 4.7
Body Mass Index*	1.02	0.96 to 1.11	−0.11030 (0.00017)	0.26	1.01	0.93 to 1.14
Metabolic syndrome	1.51	0.53 to 4.30	**0.17552** (**0.00224**)	**0.05**	2.29	0.67 to 7.77
Renal disease	0.63	0.165 to 2.30	0.06337 (0.00156)	0.52	1.83	0.85 to 3.92
Creatinine (mmol/L)*	0.99	0.99 to 1.00	0.00325 (0.00002)	0.96	0.99	0.99 to 1.00
Prior venous thromboembolism	1.73	0.62 to 4.85	−0.00251 (0.00291)	1.21	**4.16**	**1.08 to 15.9**
SDI*	1.28	0.89 to 1.85	−**0.18343** (**0.00080**)	**0.04**	**1.77**	**1.21 to 19.2**
SLEDAI*	0.94	0.77 to 1.14	0.06239 (0.00040)	0.24	**1.77**	**1.15 to 2.62**
C3*	1.44	0.0.32 to 6.51	−0.03074 (0.00351)	0.74	2.27	0.23 to 22
C4*	1.02	0.00 to 799.6	−0.17369 (0.00026)	0.05	1.74	0.5 to 5.95
Ro positive	**0.31**	**0.11 to 0.86**	−0.004072 (0.00221)	0.92	1.16	0.34 to 3.98
La positive	**0.45**	**0.13 to 1.57**	0.04982 (0.00276)	0.61	1.40	0.34 to 5.88
RNP positive	0.50	0.14 to 2.17	0.05530 (0.00310)	0.46	0.63	0.07 to 5.49
Anticardiolipin antibody positive	**3.64**	**1.27 to 10.40**	−0.08619 (0.00206)	0.45	1.46	0.23 to 4.99
Lupus anticoagulant positive	0.93	0.36 to 2.41	−0.002 (1.191)	0.41	2.11	0.35 to 12.44
Antimalarial use	0.37	0.09 to 1.53	−0.12049 (0.00274)	0.19	No events is non-use group	
Antiplatelet use	0.60	0.23 to 1.59	−0.02194 (0.00208)	0.76	0.97	0.30 to 3.06
Statin use	7.39	0.86 to 63.28	0.05112 (0.00310)	0.76	1.83	0.52 to 6.48
Steroid exposure ever	1.14	0.42 to 3.06	−0.04302 (0.00227)	0.76	1.36	0.35 to 8.2
Average steroid dose (mg)*	1.03	0.95 to 1.12	0.10877 (0.00017)	0.21	**1.14**	**1.03 to 1.26**
Total steroid dose (mg)*	1.00	0.99 to 1.00	−0.00175 (1.1×10^−4^)	0.98	0.99	0.99 to 1.00
Cyclophosphamide ever	1.93	0.44 to 8.43	−0.00134 (0.00319)	0.674	4.2*	**1.77 to 35**
Azathioprine ever	1.10	0.394 to 3.12	−0.00384 (0.00204)	0.065	3.30	0.94 to 11.58
Framingham-based 5-year CVD % risk (only patients with no prior CVD)*	**1.47**	**1.12 to 1.93**	−0.000464 (0.00034)	0.262	1.09	0.88 to 1.35

Items in bold denote significant results on univariate or age-adjusted analyses

Denotes baseline factors analysed as continuous variables.

CHD, coronary heart disease; CIMT, carotid intima–media thickness; CVD, cardiovascular disease; NA, not applicable; RNP, ribonucleoprotein; SDI, Systemic Lupus International Collaborating Clinics damage index; SLEDAI, Systemic Lupus Erythematosus Disease Activity Index.

In a multivariable analysis, lower systolic blood pressure, lower triglycerides and metabolic syndrome all remained independently associated with CIMT progression (table 5).

**Table 5:** Multivariable analysis of baseline factors associated with progression of carotid intima–media thickness (CIMT)

Baseline factors	CIMT progression	
	β (SE)	P values
Systolic blood pressure (per 10 mm Hg)	−0.00012 (0.00004)	0.004
Fasting glucose (per mmol/L)	−0.00148 (0.00095)	0.121
Triglycerides (per mmol/L)	−0.00492 (0.00138)	0.001
Metabolic syndrome (yes/no)	0.00473 (0.02102)	0.027

### Clinical outcomes

Twelve (9.7%) patients had 13 CVD events between visits (one patient had both a cerebral and coronary event) (table 6). Of the 12 (9.7%) patients with known clinical CVD at baseline, 4 (33%) had a further event. In those free of CVD at baseline (n=112), the new event rate was 7.2% (eight patients) over 5.8 years. In contrast, the median predicted 5-year percentage risk for this cohort was 1 (1–3)% (p<0.001). The median (IQR) 5-year percentage risk tended to be higher in those with a future CVD event (2.5 (1.5–5)% vs 1 (1–3)%, p=0.12).

**Table 6:** Rate of cardiovascular events (CVEs) at follow-up

	Patients with events	Rate over 5.8 years
All CVEs	12	9.7
Coronary event	7	5.6
Cerebral events	5	4
Peripheral vascular events	1	0.8
Primary CVE	8	7.2
5-year % Framingham estimate		1.0 (1.0–3.0)%

### Predictors of clinical CVD events

In an age-adjusted univariable analysis (table 4), a number of classic risk factors and SLE-related factors were associated with future CVD events. Neither presence of carotid plaque nor CIMT at baseline predicted future events (date on file). In a multivariable analysis, higher triglycerides (OR 3.61; 1.23 to 10.56), cyclophosphamide exposure ‘ever’ (OR 16.7; 1.46 to 63.5) and the SDI score (OR 9.62; 1.46 to 123) independently predicted CVD events (table 7). When we removed the CVD descriptors from the SDI, it remained in the model (OR 9.89; 1.51 to 64.4).

**Table 7:** Multivariable analysis of baseline factors associated with cardiovascular events (CVEs)

Baseline factors	CVEs	
	OR	95% CI
Triglycerides (per mmol/L)	3.61	1.23 to 10.56
Cyclophosphamide ever	16.7	1.46 to 123
SDI>0	9.62	1.46 to 63.5

SDI, Systemic Lupus International Collaborating Clinics damage index.

## Discussion

In this longitudinal study of white British women with SLE, we found over a median 5.8 years of follow-up, 26% developed new carotid plaque and carotid plaque progressed overall in 41%. In addition, 7.2% of those free of CVD at baseline had a new CVD event in this period, a sevenfold higher rate than predicted by the Framingham model.

Our data accord with the Hopkins Cohort study in which new carotid plaque was observed in 26% of patients, and at the second assessment, a higher plaque index was observed in another 17.5%.^27^ In a general population-based study of women aged 59–71 years, carotid plaque progressed in 18% over 4 years.^28^ Therefore, our findings and those of Kiani *et al* suggest that carotid plaque progresses at a higher rate in patients with SLE than would be expected.^27^ Our overall carotid plaque progression rate (4.5% per annum) is in agreement with that estimated by Thompson *et al* in Pittsburgh (6.5% per annum). In addition, like us, Thompson *et al* noted only minimal plaque regression (5%).^29^ As expected, age is a key determinant of new plaque development. Interestingly, we found that of SLE features, anti-Ro antibody was associated with reduced risk of new plaque and aCL antibodies were associated with greater plaque progression. The association with aCL antibodies remained significant in both a multivariable model and in a model including classical risk factors. We defined aCL antibodies as the presence of two positive results at a moderate to high titre at any time during the disease duration. Our previous study had found a cross-sectional association between aCL and prevalent plaque.^16^ Our current results suggest this association was with atherogenesis and may reflect the presence of cross-reacting epitopes with our clinical assay that are pro-atherogenic. Previous work has suggested that certain autoantibody subtypes such as anti-HDL or anti-oxLDL antibodies may be associated with aCL and be in themselves atherogenic.^30^ Ro-positive patients may represent a phenotypic subset with a lower risk of atherosclerosis, although the precise mechanism(s) underlying this cannot be ascertained from this study.

The mean change in CIMT in our cohort (0.002 cm/year) is in the range reported in other SLE studies (0.0012–0.0039 cm/year).^31–33^ This is approximately double the rate previously reported in the general population.^34^ In contrast, a recent controlled study has suggested that CIMT progression in SLE is no greater than a control population.^32^ In the current study, univariable factors associated with CIMT change were total cholesterol, the metabolic syndrome, SDI and C4 levels. Paradoxically, SDI was negatively correlated with CIMT progression suggesting a higher SDI correlates with less IMT progression. This may be an artefact due to the small sample size of the study, given the wide CIs observed. Another possible explanation is a surviving cohort effect, that is, those with higher rates of damage accrual have left the cohort at baseline. While the baseline SDI was comparable in those who were and were not followed, we were unable to assess if those who were lost to follow-up accrued further damage after their baseline assessment (although we do know that in this group at least six developed a new cancer and two had major cardiovascular events). Similarly, in the multivariable analysis, triglyceride level, systolic blood pressure and fasting glucose were negatively correlated with CIMT progression. Again, sample size or a surviving cohort effect may explain these apparently paradoxical results.

Our study also adds to the observations of others that clinical CVD events occur at a higher rate in SLE that is predicted by usual risk estimate equations. In this study, there was a sevenfold higher rate of cardiovascular events than predicted. In addition, the only traditional risk factor which significantly predicted events was triglyceride levels. Esdaile *et al* previously noted a sevenfold higher than predicted rate of events in a cohort of patients with SLE^4^ and Bessant *et al* also noted that while the mean 10-year percentage risk of coronary events in their cohort was 1.4 (0.2–3.4)%, over follow-up, 8.5% actually had a coronary event.^35^ With regard to risk factors for clinical events, in a longitudinal analysis of the Toronto cohort, age and triglyceride levels were the only significant predictors of future CVD events. This emphasises the need for better risk stratification models in patients with SLE. With regard to SLE-related factors, our study was underpowered to assess a range of factors. Cyclophosphamide exposure was a strong predictor of cardiovascular events at follow-up and remained in the multivariable model. While this association could reflect unmeasured confounders such as disease severity and cumulative steroid burden, the small number of patients on cyclophosphamide and wide CIs do mean this result should be interpreted with caution. However, the association between higher SDI and future CVD events both in this study and in a previous case–control study in the UK may support this hypothesis.^3^ A higher SDI is likely to reflect the cumulative burden of chronic inflammation^36^ and steroid exposure^37^ or alternatively, patients prone to damage in one organ system may also be more prone to atherosclerotic damage.^38^ Further studies investigating these associations are therefore warranted.

It is interesting to note that neither CIMT nor the presence of plaque at baseline were associated with CVD events at follow-up and that different baseline factors predicted subclinical and clinical outcomes. Both CIMT and carotid plaque have been shown to be predictors of long-term cardiovascular mortality in large population studies. Kao *et al* undertook the only published study, which has evaluated the association between baseline CIMT and plaque with future CV events in SLE.^19^ In this study of 392 female patients with SLE (median follow-up 7.9 years), a borderline association between CIMT, plaque presence and incident CV events was observed (OR (95% CI) 1.14 (1.00 to 1.31) and 1.83 (0.91 to 3.66) for baseline CIMT and plaque, respectively). When only ‘hard’ CV events were considered (angina or TIA excluded), the association became statistically significant. This study had a larger sample and followed for longer than the current study. Thus, it is likely that we were underpowered to detect the association between subclinical disease and subsequent clinical events. There is also some debate about which measure of plaque progression to use in longitudinal analyses. It should be noted that risk factors for the initiation of atherosclerosis may be different to those for disease progression or severity. In this regard, the most appropriate comparison groups may be those with no plaque versus those with new plaque over time. To obtain meaningful results, long-term follow-up of a large cohort of patients is required. The differences observed in this study with regard to CIMT may also reflect the differences in measurement as some previous studies have limited measurement to one part of the carotid artery.^31^ Additionally, unlike previous studies, the current study was limited to white British women, as the original cohort was set up to study genetic factors. This is a limitation of the current study and there is a need to conduct further CVD outcome studies in non-Caucasian populations.

A number of other limitations need to be considered when interpreting these findings. The sample size in the current study of 124 patients is relatively small and while the average follow-up period of more than 5 years is longer than in previously reported studies, this is still a relatively short period of follow-up. The limitations of sample size and duration of follow-up in this study may have influenced the ability to detect a correlation between clinical events and subclinical measures. Furthermore, the clinical events were patient reported, which is a weakness. We also acknowledge that our follow-up cohort only included 62% of our original group. We made every reasonable effort to contact all patients; however, a number were truly lost to follow-up, which may be inevitable in a mobile urban population. In addition, another large group declined to return for study, mainly due to changing of their personal circumstances. There is of course the potential for bias being introduced. When examining differences in baseline characteristics of those followed or lost to follow-up, lower serum triglyceride levels and higher systolic blood pressure were noted in those who were followed up. All other CVD risk factors and disease characteristics were similar between the groups. Those lost to follow-up did not have any consistent markers of a differential level of disease severity or CVD risk. However, if those lost to follow-up had more severe disease, it would, if anything, tend to bias us towards a conservative estimate of clinical and subclinical disease progression. Whether or not this is the case, the factors we identified as significant predictors will maintain validity within this cohort and since our power will be limited by the smaller follow-up group, significant results remain of relevance. However, we acknowledge that several important factors (eg, antimalarial use) may have been identified or confirmed, due to limited power.

Longitudinal studies provide the opportunity to determine factors that will help us improve risk stratification of SLE populations for future CVD events and the identification of potentially modifiable risk factors. Our results suggest that a more comprehensive approach to risk stratification is needed as well as classic risk factors, for example, triglycerides which are less often a target for intervention also contribute to future CVD events in SLE. A higher-risk population may also be identified from patients who require potent immunosuppression as well as those with anticardiolipin antibodies. Such factors may be a useful adjunct to routine CVD screening approaches and may result in improved CVD risk in this high-risk population.
